# Bio-distribution study of Tc-99m HMPAO labeled platelet in healthy volunteer

**DOI:** 10.22038/AOJNMB.2024.71620.1525

**Published:** 2024

**Authors:** Mahdieh Parvizi, Mehrshad Abbasi, Hojjat Ahmadzadehfar, Abbas Tafakhori, Maryam Naseri, Ali Khalaj, Saeed Farzanehfar

**Affiliations:** 1 Department of Radiopharmacy, Faculty of Pharmacy, Tehran University of Medical Sciences, Tehran, Iran; 2 Department of Nuclear Medicine, Imam Khomeini Hospital Complex, Tehran University of Medical Sciences, Tehran, Iran; 3 Department of Nuclear Medicine, University Hospital Bonn, Bonn, Germany; 4 Department of Neurology, School of Medicine, Imam Khomeini Hospital Complex, Tehran University of Medical Sciences, Tehran, Iran; 5 Department of Medicinal Chemistry, Faculty of Pharmacy, Tehran University of Medical Sciences, Tehran, Iran

## Abstract

**Objective(s):**

The bio-distribution of Tc-99m HMPAO labeled platelets (LP), which could be used to image subtle thrombosis, is not reported in a human yet, which is the subject of the current study.

**Method:**

The platelets were extracted from 49 ml whole blood and labeled with Tc-99m HMPAO, then re-injected to the healthy volunteer. Anterior and posterior whole body imaging was done by a dual-head gamma camera 3, 18, 33, 46, 81, 124, 190 min and 15 hours after injection. Also a whole-body SPECT was done at 137 min post-injection. The area under the curves of the spleen, liver, left kidney, bladder, right lung, brain, and abdominal aorta ROIs was calculated to estimate the accumulation of labeled platelets within the organs.

**Results:**

The spleen was the target organ. The kidneys, liver, and heart were also remarkably visualized. The thyroid, stomach, bladder, or gastrointestinal (GI) uptake/activity was not significant. The stomach visualization was enhanced after ingestion at 60 min. The sagittal and lateral sinuses were delineated, and the background of the brain was very low. During the study, the area under the curve of activity was 738, 308, 302, 196, 230, 121, 79, 216, 529, 369, 162, and 54 counts. min/pixel for spleen, liver, heart, right lung, left kidney, right iliac artery, sagittal sinus, thyroid, bladder, stomach, GI, and background, respectively.

**Conclusion:**

The quality of the scan with low dose Tc-99m HMPAO LPs is optimal. We documented the bio-distribution of LPs. The optimal imaging time was 80-120 min post-injection when the free Tc-99m and GI transit were negligible. The sagittal and lateral sinuses were visualized enabling detection of possible clots in the vessels.

## Introduction

 There are multiple clinical implications for imaging of the thrombosis including radio-labeled monoclonal antibodies that have been applied to target fibrin on acute thrombosis in humans (1, 2), but they had very limited clinical use due to their long blood circulation time. 

 Synthetic Tc-99m labeled peptides like aptitude, a glycoprotein (GP IIb/ IIIa receptor antagonist), and DMP 444, a platelet IIb/IIIa antagonist, was increasingly used as a specific tracer for detection of acute deep vein thrombosis (DVT). But due to their non-availability in certain countries, low target to background ratio secondary to slow blood clearance, and high cost of the tracers, these scans did not find their place in routine clinical practice (3, 4). One needs to resort to other tracers for thrombosis detection (5-13). In 1976 Thakur et al., for the first time, reported labeling platelets with In-111 hydroxyquinoline. Several studies demonstrated the value of platelet scintigraphy as an investigative tool for monitoring platelets function (14), but the image quality of In-111 was not optimal while the availability was limited. Over the years, Tc-99m-hexamethylpropyleneamine oxime (Tc-99m-HMPAO) has been used instead of In-111 to label the platelets due to its availability, appropriate physical characteristics, higher image quality, lower cost, and less radiation dose (15,16). The lower effective dose from Tc-99m permits the use of higher injection doses and consecutive better image quality enabling imaging smaller clots (15). We standardized the platelet extraction procedure and labeling with Tc-99m-HMPAO (17) and demonstrated its bio-distribution and image quality in the rabbit with induced clot (18). We are determined to use the labeled platelet imaging to answer certain clinical questions. One would be imaging of clots in cerebral venous sinus thrombosis to differentiate the etiology for pseudotumor cerebri. Before clinical application, in the current study, we present the bio-distribution and imaging in a healthy human, a volunteer subject one of the authors.

## Methods

**Figure 1:**
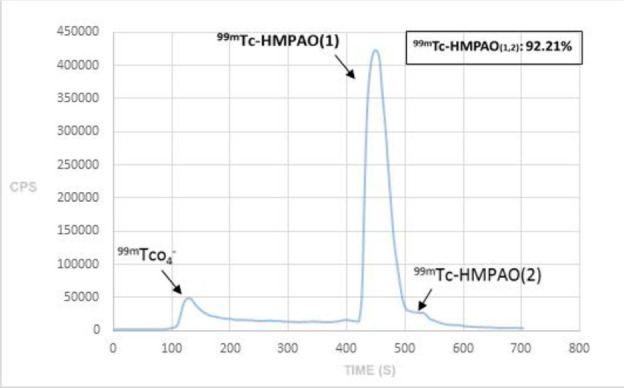
The TLC of Tc HMPAO preparations

 The platelets were extracted as explained before (17). In brief, 49 ml whole blood was withdrawn from the catheterized antecubital vein in a 60 ml syringe containing 11 ml (ACD; Acid Citrate Dextrose, Pars Isotope Co, Tehran, Iran). The blood and ACD were mixed with a gentle shake and split into two 50 ml falcon tubes and centrifuged at 1600 RPM for 15 min. The platelet-rich plasma (PRP) was separated carefully without red blood cell (RBC) contamination with a volume of about 29 ml and centrifuged again at 2500 RPM for 15 min. Platelet-poor plasma was removed and stored at 37 ˚C for the next steps and final injection. Platelet pellet was collected at the bottom of the falcon tubes and re-suspended in ACD and saline (0.5 ml: 3 ml). An HMPAO kit (Pars Isotope Co, Tehran, Iran) was labeled by 5 ml of 2516 MBq (68 mCi) Tc-99m from the freshly eluted generator. The labeling process was simple with 2 min shaking. The TLC radio-chromatogram for radiochemical analysis of Tc HMPAO is presented in Figure 1. The content of the HMPAO kit was dropped wisely added into the re-suspension of the platelet extraction in ACD and saline and incubated for 25 min at 37 ˚C. After the addition of 4 ml PPP, the third round of centrifugation was done at 1700 RPM for 10 min. The supernatant containing unbound Tc-99m HMPAO was discarded and the platelet pellet labeled with 148 MBq (4 mCi) Tc-99m was again resuspended in 3 ml PPP. The final preparation at a total volume of about 4-5 ml was re-injected to the catheterized antecubital vein and flushed with 5 ml normal saline.

Imaging was done by a dual-head gamma camera (Anyscan, mediso, Budapest, Hungary) in the Department of Nuclear Medicine. Three min after injection, first whole-body imaging at anterior and posterior projections was done for 13 min at 15 cm/min speed followed by similar whole-body scans at 18, 33, 46, 81, 124, and 190 min and 15 hours. The 124 min delayed whole-body image duration was 15 min 50 sec. A whole-body (i.e. head and neck, thorax, and abdominopelvic) SPECT was done at 137 min post-injection. The following specifications were used: matrix size 128×128, 1.14 zoom in 64 views, and 25 second projection time.

For quantitative analysis, the region of interest (ROIs) were drawn over the spleen, liver, left kidney, bladder, right lung, brain, and abdominal aorta. The area under the curves (AUCs) of the geometric mean of anterior and posterior ROIs was calculated to estimate the accumulation of labeled platelets within the organs.

## Results

**Figure 2:**
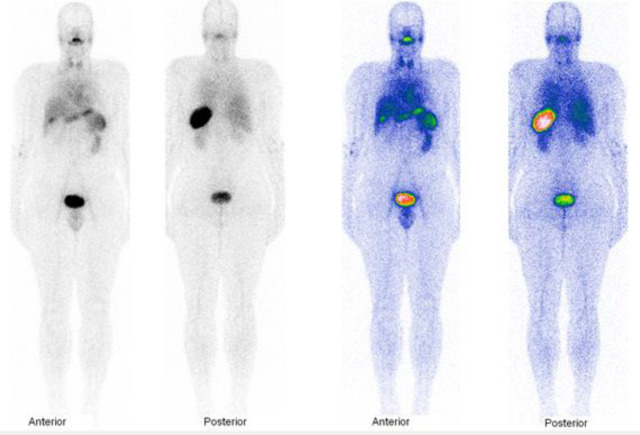
Whole-body images of Tc-99m-HMPAO labeled platelet; the image of healthy 95 kg volunteer 81 min (no anesthesia employed) after injection of 148 MBq (4 mCi) Tc-99m-HMPAO labeled self-donated platelet. Image acquisition was done for 13 min at 15 cm/min speed

**Figure 3:**
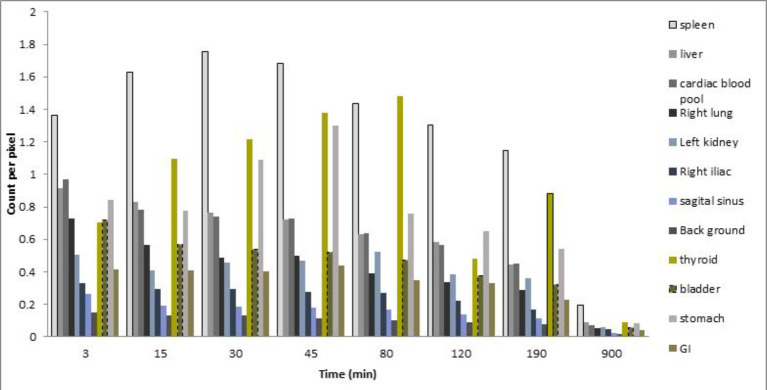
The quantification of activity within the organs (no decay correction employed)

**Figure 4:**
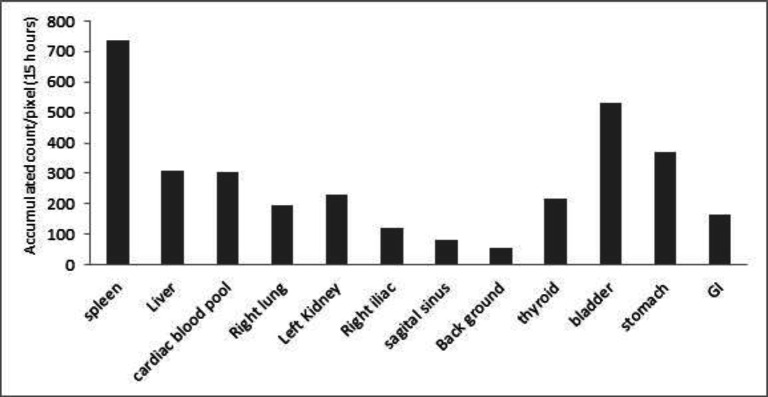
Area under the curve of activity within the organs up to 900 min after injection of 148 MBq (4 mCi) Tc-99m-HMPAO labeled platelet

 The planar images illustrated in Figure 2 show accumulation of the tracer within the spleen, liver, lung, heart, and blood pool, thyroid, and kidney; the activities within the organs are presented in Figure 3. AUC of the activity accumulation within the ROIs of target and interesting organs in the 15-hour post-injection were 738.3, 308.2, 301.8, 195.8, 229.7, 120.9, 79.3, 215.5, 528.9, 368.7, 162.3, and 53.7 counts. min/pixel for spleen, liver, cardiac blood pool, right lung, left kidney, right iliac, sagittal sinus, thyroid, bladder, stomach, gastrointestinal (GI), and background, respectively (Figure 4). The spleen is the target organ with peak activity at 15 to 45 min images. The next organs with the highest tracer accumulation were the liver and cardiac blood pool with decreasing activity during the study. The activity of kidneys and bladder was visualized at early image right after injection and these organs remained visible with the same activity up to the end of the study. 

The activity in the thyroid and stomach was noticed early post-injection with a peak at 45 to 80 min and 30 to 45 min after injection, respectively. A cup of sweet tea was taken at 60 min with remarkable stomach visualization afterward. Otherwise, the GI transit of the activity was not considerable and stable up to the end of the study. In a 15-hour delayed image, the spleen was still the target organ with tracer visualization in the GI tract and bladder without proper visualization of the blood pool. 

**Figure 5:**
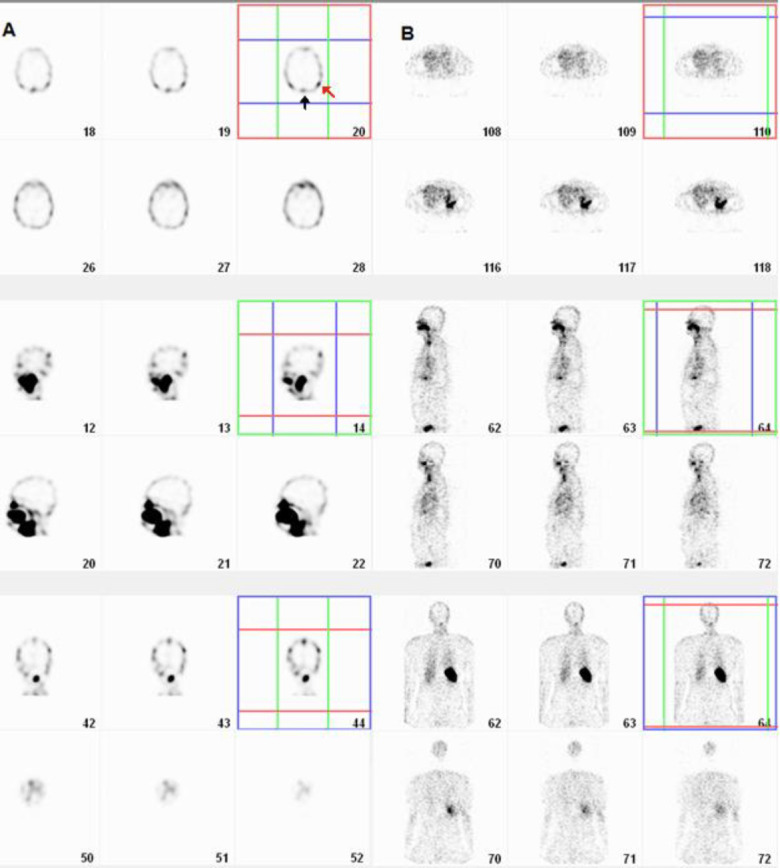
SPECT of the head and whole-body SPECT after injection of 148 MBq (4 mCi) Tc-99m-HMPAO labeled platelet; the sagittal and transverse sinuses (**black and red arrows**) are perfectly visualized

 In the whole-body SPECT (Figure 5), the spleen, liver, lungs, and abdominal main vessels and cardiac blood pool were visualized. The SPECT of the head illustrates the vessels delicately so that the cerebral sagittal sinus and transverse sinuses were delineated (Figure 5; arrows). As an index for the optimal visualization of smaller vessels the ratio of the activity in the right iliac artery to the liver was calculated 0.36, 0.36, 0.38, 0.39, 0.43, 0.38, and 0.37 at 3, 18, 33, 46, 81, 124, and 190 min images, respectively; which peaked at 81 min.

 The volunteer participant experienced no adverse effect during or after the examination until the date of this publication. 

## Discussion

 Tc-99m-HMPAO labeled platelets were safe in our experience with good imaging qualities. The procedure was done with the self-donated whole blood of about 49 ml and 2 hours were required for the process before the injection. 

 We suggest the optimal imaging time would be between 1 and 2 hours after injection while the patient is fasting. At this time interval, not only does the blood pool to background activity become optimized but also the GI transit does not interfere with the blood pool of the main vessels in the abdomen. Although, the thyroid and stomach is visualized (Figure 1), the AUC of the thyroid was lower and stomach comparable with AUC of blood pool which indicates that free technetium level is not high similar to the situations when thyroid and stomach uptake is higher than blood pool e.g. thyroid scan. The uptake in the area under the question of brain, sagittal sinus, was comparable to back ground which indicates very low level of free Tc-99m HMPAO which is brain perfusion imaging with high brain uptake.

 The activity of 148 MBq (4 mCi) was employed, and the image quality was acceptable; had we administered higher activities up to 740 MBq (20 mCi) similar to those used in the RBC tagged scan, the image quality would have been enhanced. The higher doses might be plausible in clinical cases when a small clot should be imaged.

 With particular attention to the brain and cerebral sinuses, the uptake of the tracer within the brain was low and the blood pool in the sagittal and transverse sinuses was relatively high. We have already documented that a clot with a 6 mm diameter was successfully imaged in the rabbit with the clinical gamma camera. 

 We have also shown that the procedure of platelet extraction is feasible and repeatable in clinical settings. In an ordinary nuclear medicine center, the only device to be added is a centrifuge. Collaboration with a pathology facility and knowledge for quality control are also needed. The possible failures of the procedure are the use of old HMPAO kits, wrong centrifuge speed, and unadjusted PH; fast shaking results in cell lysis. The operator needs to repeat the procedure a few times before initializing the clinical usage with close supervision to prevent contamination and preserve sterility.

 There are many approaches for detection of the thrombosis including Doppler ultra-sonography (19), computed tomography (CT) angiography (20), magnetic resonase (MR) angiography (21), perfusion imaging with macro-aggregated albumin (22), and clot detection with specific anti-platelet antigen antibodies (23). There are limitations for any currently available thrombus imaging method: the extremity or calf ultrasonography is not accurate for the pelvic veins, the source of the most pulmonary thromboembolism. 

 Anatomical imaging such as CT and MRI are used as initial neuroimaging tests in patients who present with new-onset neurological symptoms such as a headache, seizure, and mental alteration. With an emphasis on cerebral sinus thrombosis, the anatomic variability of the venous sinuses makes a diagnosis using CT insensitive (24). Other techniques including intravascular ultrasound, brain angiography, and direct cerebral venography via catheterization are the standard methods in many conditions and can provide anatomic details of plaque size and composition. Although these techniques have the disadvantage of being invasive and demanding, hence, are less commonly clinically indicated (4). CT and MR angiography have the potential for contrast-induced nephropathy (25) and have limited accuracy for thrombosis detection within cerebral veins or in particular conditions including in post-transplant patients. 

 Furthermore, certain thrombosis including the cerebral vein thrombosis is essentially less possibly detectable by anatomical imaging comprising MRI. In contrast, nuclear medicine techniques have the potential to assess thrombosis formation. Radiolabelled mono-clonal antibodies have been used to target fibrin, but as the result of long blood circulation time, they had limited clinical usage. Also, antibody tracers provoke autoimmune process. 

 The labeled macro-aggregated albumin is a cold spot imaging with naturally low contrast. 

 Labeled fibrin imaging is applicable in acute phases of the thrombosis (26) and the accuracy of Tc-99m apcitide and DMP 444 scans were low and had limited utility for imaging patients with pulmonary thromboembolism (27). Tc-99m HMPAO labeled platelet scan has the potential to fit the gap of these imaging technics and promote thrombosis imaging. For example, in pseudotumor cerebri, Tc-99m-HMPAO labeled platelet scan may assist the neurologist because the labeled platelets directly insert into the thrombus. Based on current results, we are determined to use this scan for the detection of clots in the cerebral sinus of patients suspected for the diagnosis of cerebral venous sinus thrombosis.

## Declarations

### Ethics approval and consent to participate

 This study has been approved by the Tehran University of Medical Sciences ethics committee and has therefore been performed in accordance with the ethical standards laid down in the 1964 Declaration of Helsinki and all subsequent revisions. The participant’s written informed consent form was collected before the study.

 Informed consent was obtained from all individual participants included in the study.

## Data Availability

The data is available on request email to corresponding author.
